# Editorial: Addressing fracture risk in aging populations: integrated prevention tactics

**DOI:** 10.3389/fpubh.2026.1812896

**Published:** 2026-03-12

**Authors:** Mohammad Daher, Amer Sebaaly

**Affiliations:** Hotel Dieu de France, Beirut, Lebanon

**Keywords:** bone health, fragility fracture, osteoporosis, public health, sarcopenia

## Introduction

Fragility fractures are increasingly prevalent in the aging population due to factors like osteoporosis and diminished bone strength, posing significant challenges to global health systems in terms of morbidity, mortality, and economic cost (1–3). Addressing factors such as osteoporosis is crucial as the latter can also affect the surgical outcomes in various orthopedic surgeries (4–12). However, preventing these fractures remains the ideal approach. The aim of this Research Topic is to investigate and improve prevention strategies by employing a comprehensive, interdisciplinary approach that incorporates insights from epidemiology, gerontology, orthopedics, and health policy.

This Research Topic comprises 14 articles spanning epidemiologic analyses, cross-sectional and cohort studies, scoping reviews, meta-analyses, predictive modeling studies, and machine-learning–based investigations. Most contributions originate from China while also including global analyses and hospital-based investigations, reflecting both population-level and clinical perspectives. The articles are organized into three thematic categories: (1) epidemiology, disparities, and prediction of fragility fractures; (2) outcomes; and (3) factors affecting bone and muscle health.

## Epidemiology, disparities, and prediction of fragility fractures

This section highlights emerging evidence on the epidemiology and inequities of fragility fractures alongside advances in predictive analytics that collectively refine identification of high-risk older adults and inform targeted prevention strategies. At the population level, Gu et al. demonstrated substantial geotemporal disparities in hip fracture burden among adults aged ≥55 years across 204 countries, showing that global age-standardized incidence and prevalence increased between 1990 and 2021 and are projected to continue rising through 2050, largely driven by population aging and growth, with disproportionate burden in high-SDI regions and a more pronounced increase among males. Similarly focusing on disparities, Lin et al. examined urban–rural differences in fall risk among older Chinese adults, reporting an overall fall incidence of 22.4%, higher in rural than urban populations, and showing that while common predictors included sex, age, prior falls, sleep duration, functional status, memory, and chair stand performance, the relative importance of these factors differed by setting. Complementing these epidemiologic insights, Diep et al. identified a fall prevalence of 19.6% among hospitalized older adults and found that prior falls and osteoarthritis were the strongest predictors, reinforcing the role of targeted screening. Moving toward individualized prediction, Li and Shi applied machine-learning models to forecast hip fracture incidence, identifying key predictors such as metabolic equivalent of task, age, alcohol use, cognitive scores, sleep patterns, residence, and marital status. Likewise, Kim et al. developed explainable AI models for frailty prediction that highlighted depression score, age, instrumental activities of daily living, sleep quality, and cognition as dominant predictors within a multidimensional framework spanning functional, behavioral, and social domains. Finally, focusing on secondary prevention, Qi et al. constructed a nomogram to predict re-fracture risk in older adults with osteoporotic vertebral fractures, identifying tumor history, scoliosis, mental disorders, prolonged alcohol use, and chronic kidney disease as significant predictors.

## Outcomes

This section synthesizes evidence on post-fracture outcomes across the care continuum highlighting opportunities to strengthen services and improve functional and health trajectories after fragility fractures. Zhu and Ran examined outcomes and care needs among rural Chinese older adults following osteoporotic fractures, reporting a fracture prevalence of 58.7%, with half of affected individuals experiencing life-impacting consequences. Their findings underscored marked socioeconomic disparities, as insured individuals and homeowners were more likely to receive post-fracture assistance, while limited policy awareness and unmet needs for governmental support represented major barriers; medical interventions such as supplement use and osteoporosis treatment were associated with higher odds of receiving help, whereas family-provided support sometimes reduced access to formal services (Zhu and Ran). Expanding on continuity of care, Zhang et al. synthesized evidence from 17 studies and found that demand for ongoing care after hip fracture ranged from 35.83 to 75.60%, encompassing five domains: access to hospital and community resources, disease-related knowledge, social support, nutrition, and psychological needs. Among these, access to services and informational needs were most prominent, with demand influenced by socio-demographic factors and clinical characteristics (Zhang et al.). Finally, focusing on acute postoperative outcomes, Kang et al. evaluated perioperative predictors of delirium in geriatric hip fracture patients and identified higher admission rate pressure product and elevated preoperative interleukin-6 levels as independent risk factors.

## Factors affecting bone and muscle health

This section explores modifiable determinants of bone and muscle health, integrating evidence on physical fitness, exercise dosing, nutrition, and imaging-based analytics to clarify links between sarcopenia, bone mineral density (BMD), and fracture risk. Wei et al. reported positive associations between grip strength and BMD, with quadriceps strength correlating with BMD in women and maximal oxygen uptake correlating with BMD in men. Supporting exercise as a therapeutic strategy, Wang Y. et al. (meta-analysis) found aquatic training significantly improved muscle strength, with optimal benefits at ~24 weeks, 2–3 sessions weekly, and ~100 min per week. Addressing nutritional and metabolic influences, Wang X. et al. further showed that vitamin D, psoas muscle index, and femoral neck BMD independently protected against hip fracture, with muscle metrics mediating up to half of vitamin D's effect, while Shen and Wei demonstrated a linear association between higher low-carbohydrate diet scores and increased osteoporotic fracture risk. Finally, focusing on measurement innovation, Zuo et al. showed that unsupervised CT- and MRI-based machine-learning models accurately predicted sarcopenia, with CT-based algorithms achieving excellent performance (AUC up to 0.99), highlighting their potential for early risk detection.

Despite the breadth of contributions, several important gaps remain. Most studies were conducted in Asian populations, highlighting a need for broader geographic representation, particularly from low- and middle-income regions outside East Asia. Interventional and randomized controlled studies remain limited, with much of the current evidence derived from observational or predictive modeling designs. Additionally, vulnerable subgroups such as the very old (≥85 years), institutionalized individuals, and ethnically diverse populations remain comparatively underrepresented. Future research should prioritize implementation science approaches and culturally adaptable prevention strategies to ensure equitable global applicability.

## Conclusion

Collectively, the findings of this research topic carry significant public health implications. The consistent identification of modifiable risk factors supports the integration of musculoskeletal health screening into primary care and community-based aging programs. Advances in machine-learning and explainable AI models offer opportunities for scalable, cost-effective risk stratification tools that can be embedded within public health systems to proactively identify high-risk individuals. Moreover, the demonstrated disparities in fracture burden and post-fracture care access underscore the necessity of policy-level interventions aimed at improving insurance coverage, rural healthcare access, patient education, and continuity of care services. An integrated strategy that bridges epidemiologic surveillance, early risk prediction, equitable service delivery, and targeted preventive interventions will be essential to mitigating the projected rise in fragility fractures globally.
